# Air Displacement Plethysmography versus Dual-Energy X-Ray Absorptiometry in Underweight, Normal-Weight, and Overweight/Obese Individuals

**DOI:** 10.1371/journal.pone.0115086

**Published:** 2015-01-21

**Authors:** David W. Lowry, A. Janet Tomiyama

**Affiliations:** 1 School of Medicine, Case Western Reserve University, 10900 Euclid Ave., Cleveland, Ohio 44106, United States of America; 2 Department of Psychology, University of California Los Angeles, 1285 Franz Hall, 502 Portola Plaza, Los Angeles, California 90095-1563, United States of America; NIH / NIDDK, UNITED STATES

## Abstract

**Background:**

Accurately estimating fat percentage is important for assessing health and determining treatment course. Methods of estimating body composition such as hydrostatic weighing or dual-energy x-ray absorptiometry (DXA), however, can be expensive, require extensive operator training, and, in the case of hydrostatic weighing, be highly burdensome for patients. Our objective was to evaluate air displacement plethysmography via the Bod Pod, a less burdensome method of estimating body fat percentage. In particular, we filled a gap in the literature by testing the Bod Pod at the lower extreme of the Body Mass Index (BMI) distribution.

**Findings:**

Three BMI groups were recruited and underwent both air displacement plethysmography and dual-energy x-ray absorptiometry. We recruited 30 healthy adults at the lower BMI distribution from the Calorie Restriction (CR) Society and followers of the CR Way. We also recruited 15 normal weight and 19 overweight/obese healthy adults from the general population. Both Siri and Brozek equations derived body fat percentage from the Bod Pod, and Bland-Altman analyses assessed agreement between the Bod Pod and DXA. Compared to DXA, the Bod Pod overestimated body fat percentage in thinner participants and underestimated body fat percentage in heavier participants, and the magnitude of difference was larger for underweight BMI participants, reaching 13% in some. The Bod Pod and DXA had smaller discrepancies in normal weight and overweight/obese participants.

**Conclusions:**

While less burdensome, clinicians should be aware that Bod Pod estimates may deviate from DXA estimates particularly at the lower end of the BMI distribution.

## Introduction

Accurately estimating body composition, and in particular fat mass, is vital for assessing the health of an individual and deciding on treatment course. Hydrostatic weighing, considered the “gold-standard” measure of adiposity [1], is a highly burdensome method for both researchers and patients [2], requiring underwater submerging of the patient. Moreover, the very individuals whose treatments might benefit most from body composition or adiposity information (such as those suffering from wasting diseases or obesity type II) are those for whom hydrostatic weighing is most difficult to administer.

Due to these constraints, researchers commonly rely on dual-energy x-ray absorptiometry (DXA) to measure body composition with high accuracy [1]. DXA capitalizes on the differential attenuation of x-rays when passing through bone, lean tissue, and fat to estimate the relative mass of each. This method is highly precise and provides estimates that can rival and sometimes exceed the precision of hydrostatic weighing [3].

Although less burdensome than hydrostatic weighing, DXA exposes patients to radiation and requires specialized training and certification to operate. The goal of the current study was to evaluate air displacement plethysmography (ADP) via the Bod Pod [4], a less burdensome, less expensive, and more easily operated method of estimating adiposity than DXA that potentially sidesteps these concerns.

ADP estimates body density by deriving body volume and measuring body mass [4]. It derives body volume by capitalizing on the relationship between pressure and volume. The Bod Pod then calculates body fat percentages from body density estimates using equations proposed by Siri [5] and Brozek and colleagues [6]. Extensive details on the exact methodology of ADP and the Bod Pod, including its physical design, are available elsewhere [4] and from the manufacturer. Most importantly, the Bod Pod involves minimal participant burden. The patient simply sits in the Bod Pod, breathes through a tube, and provides three short bursts of exhalations. Furthermore, any individual can be trained to operate the Bod Pod, and the costs associated with each test run are minimal.

The specific purpose of this study was to evaluate whether the Bod Pod, in comparison to DXA, provided similar estimates in populations that are at the relative extremes of Body Mass Index (BMI; weight in kilograms/height^2^ in meters). We did so for two reasons. First, adiposity information can be particularly significant clinically in these populations by assessing the degree of underweight or wasting and tracking change in adiposity during treatment (whether that treatment is for weight loss or weight gain). Individuals at the low extremes of BMI, such as those suffering from anorexia nervosa, as well as those at the upper extreme, such as individuals with obesity type II, are examples of populations that could be assessed using the Bod Pod. Second, while the Bod Pod has been validated in a number of studies for normal weight populations [7,8], much of the research focus in validating the Bod Pod has been in obese populations [9,10]. Researchers have noted the urgent need to study Bod Pod estimates in lean individuals in particular. For example, Vescovi and colleagues [11] called for studies of the Bod Pod in individuals with fat percentages below the average fat percentage range. This study fills that gap in the literature.

## Methods

The University of California, San Francisco (UCSF) Committee on Human Research approved all procedures, and all participants provided written informed consent. All procedures took place at the UCSF Clinical and Translational Science Institute (CTSI) Clinical Research Center (CCRC). All participants were non-smokers, and no participants were pregnant. All were free of major medical diagnoses, confirmed by the CCRC Medical Director. We recruited individuals at the lower BMI extreme (*n* = 30) from the Calorie Restriction (CR) Society and followers of the CR Way. We also recruited normal weight (BMI 18.5–24.99) individuals (*n* = 15) and overweight/obese (BMI 25+) individuals (*n* = 19) from the general population. Nursing staff verified BMI on-site. We recruited the comparison groups to match the CR group in age, ethnicity, gender, and educational attainment. We additionally recruited siblings (*n* = 6; 1 overweight and all others normal weight) of CR participants to attempt to control for genetic and environmental factors. Data collection was planned before both tests were performed. Blinding staff to the BMI condition was impossible, but all Bod Pod staff were blind to the results of the DXA and vice versa. The Bod Pod and DXA measurements occurred less than 6 hours apart, and all occurred after a minimum of 2 hours post-feeding. No adverse events occurred.

## Measures

**BMI**. Nursing staff conducted measures of body weight using a Scaletronix standing scale (White Plains, NY) with participants wearing light clothing. Body height was obtained from duplicate measures using a wall-mounted stadiometer. We used these measures to calculate BMI.

**Bod Pod**. The BOD POD Gold Standard Model 2007A (Life Measurement Instruments/COSMED) using software version 5.2.0 is installed in a room within a room on the CCRC to minimize potential error due to airflow between doors and windows. Operators received all necessary training from Life Measurement Instruments training staff and the Director of the Body Composition, Exercise, and Metabolism Core for the Clinical Research Service of the CTSI. We calibrated the Bod Pod daily using a known volume (50 L) and conducted a second calibration immediately prior to the participant session. The Bod Pod weight scale was calibrated weekly. To minimize potential error due to isothermal air trapped in clothing and hair, all participants wore bathing suits or tight-fitting athletic gear and swim caps. Participants were also asked to void their bladder to minimize potential error due to excess water volume. We followed the standard recommended Bod Pod protocol, and repeated testing until merit value was less than 1. The Bod Pod generally has good repeatability of measurement, with high test-retest coefficients in past studies (e.g., 0.99 [11]; 0.99 [12]) and small coefficients of variation (2.0%-2.3% between days; 1.7%-4.5% within days [7]).

**DXA**. Certified staff from the CTSI Clinical Research Services Body Composition, Exercise, and Metabolism Core conducted whole-body DXA scans. Scans were conducted on a Lunar Prodigy DXA scanner (GE Healthcare) following standard protocols and running enCORE software version 12.3.

### Statistical Methods

We conducted all analyses using SPSS version 21.0 (IBM Corp, Armonk, NY). We obtained body fat percentages from the Bod Pod using both the Siri and Brozek equations [5,6]. To test whether body fat percentage obtained by the Bod Pod and DXA statistically differed from one another, we conducted paired-sample t-tests comparing body fat percentage estimates from Bod Pod-Siri and DXA as well as Bod Pod-Brozek and DXA. Following the recommendations of Bland and Altman [13] for assessing agreement between two diagnostic measures, we also created Bland-Altman plots comparing Bod Pod estimates to DXA. To do so, we calculated the average difference between these Bod Pod estimates and DXA as well as the standard deviation of this difference. From these means and standard deviations, we calculated 95% confidence intervals using Mean +/- 2*SD. Also following the recommendations of Bland and Altman [13], we calculated the coefficient of reproducibility as 100 * SD(Bod Pod—DXA) / ((mean Bod Pod + mean DXA)/2).

## Results

The entire sample had a mean age of 55.01(*SD* = 14.53, *min-max* = 21–84), was 78.3% male, and 88.6% white, 11.4% Asian/Asian-American, and had the following distribution of highest level of educational attainment: 3% high school, 12.1% some college, 24.2% bachelor’s degree, 21.2% master’s degree, 39.4% doctoral degree. For participants with complete data on the DXA and BodPod variables, the CR group had a mean age of 54.64 (*SD =* 15.38, *min-max* = 25–84), was 75.9% male, and 90% white, 10% Asian/Asian-American; the normal weight group had a mean age of 49.33 (*SD =* 12.49, *min-max* = 21–67), were 66.7% male, and 86.7% white, 33.3% Asian/Asian-American; and the overweight/obese group had a mean age of 58.84 (*SD =* 13.98, *min-max* = 23–80), were 88% male, and 94.7% white, 5.3% Asian/Asian-American. Descriptive statistics for BMI and the respective fat percentage estimates are displayed in Table 1. Five participants from the CR group refused DXA due to radiation concerns, and all analyses reflect those with complete data. There were no outliers (defined as greater than 3 standard deviations from the mean). The Bod Pod and DXA intraclass coefficient value was 0.90 (single measures; using a two-way mixed model and absolute agreement).

**Table 1:** Descriptive statistics of main variables.

	Underweight (n = 30)	Normal Weight (n = 15)	Overweight/Obese (n = 19)
Body Mass Index	**17.25** (1.01) 15.37–18.36	**21.53** (2.16) 18.51–24.96	**29.03** (3.38) 25.10–35.92
Bod Pod Body Fat %—Siri	**16.15** (6.47) 8.10–30.30	**22.45** (8.43) 10.70–47.50	**32.92** (8.46) 10.50–48.90
Bod Pod Body Fat %—Brozek	**16.16** (5.97) 8.75–29.23	**21.96** (7.78) 11.11–45.10	**31.64** (7.81) 10.96–46.39
DXA Body Fat %	**8.82** (7.17) 4.10–24.80	**20.02** (8.04) 6.30–41.60	**34.38** (8.43) 17.60–54.90

Note: **Mean** (Standard Deviation) Minimum-Maximum.

### Paired-sample t-tests

In the underweight group, both the Bod Pod Siri (*t* = 4.83, *p* = .001, *95% CI* = 3.62–9.96) and Brozek (*t* = 4.97, *p* = .001, *95% CI* = 3.73–9.96) body fat percentage estimates were statistically significantly higher than the DXA estimate. In the normal weight group, both the Bod Pod Siri (*t* = 2.87, *p* = .009, *95% CI* = 0.66–4.09) and Brozek (*t* = 2.34, *p* = .03, *95% CI* = 0.22–3.59) estimates were significantly higher than the DXA estimate, but the magnitude of the difference was much smaller. In the overweight/obese group, both the Bod Pod Siri (*t* = −2.46, *p* = .022, *95% CI* = −3.09–6.95) and Brozek (*t* = −4.27, *p* <. 001, *95% CI* = −4.36–−1.51) body fat percentage estimates were significantly lower than the DXA estimate.

### Bland-Altman analyses

The coefficient of reproducibility of the Bod Pod Siri estimate was 19.94%, and 21.62% for the Bod Pod Brozek estimate. For the underweight group, the Bod Pod compared to DXA provided on average 6.79% higher body fat (*SD* = 4.43, *min-max* = 0.7–13.2) for Siri estimates and 6.84% (*SD* = 4.35, *min-max* = 0.51–12.91) higher body fat using Brozek estimates. For the normal weight group, the Bod Pod compared to DXA provided on average 2.36% (*SD* = 4.05, *min-max* = −5.20–8.90) difference in body fat for Siri estimates and 1.90% (*SD* = 3.99, *min-max* = −5.87–8.81) difference in body fat using Brozek estimates. For the overweight/obese group, the Bod Pod compared to DXA provided on average 1.68% (*SD* = 3.27, *min-max* = −7.10–5.00) lower body fat for Siri estimates and 2.94% (*SD* = 3.30, *min-max* = −8.51–3.99) lower body fat using Brozek estimates.

**Figure 1:**
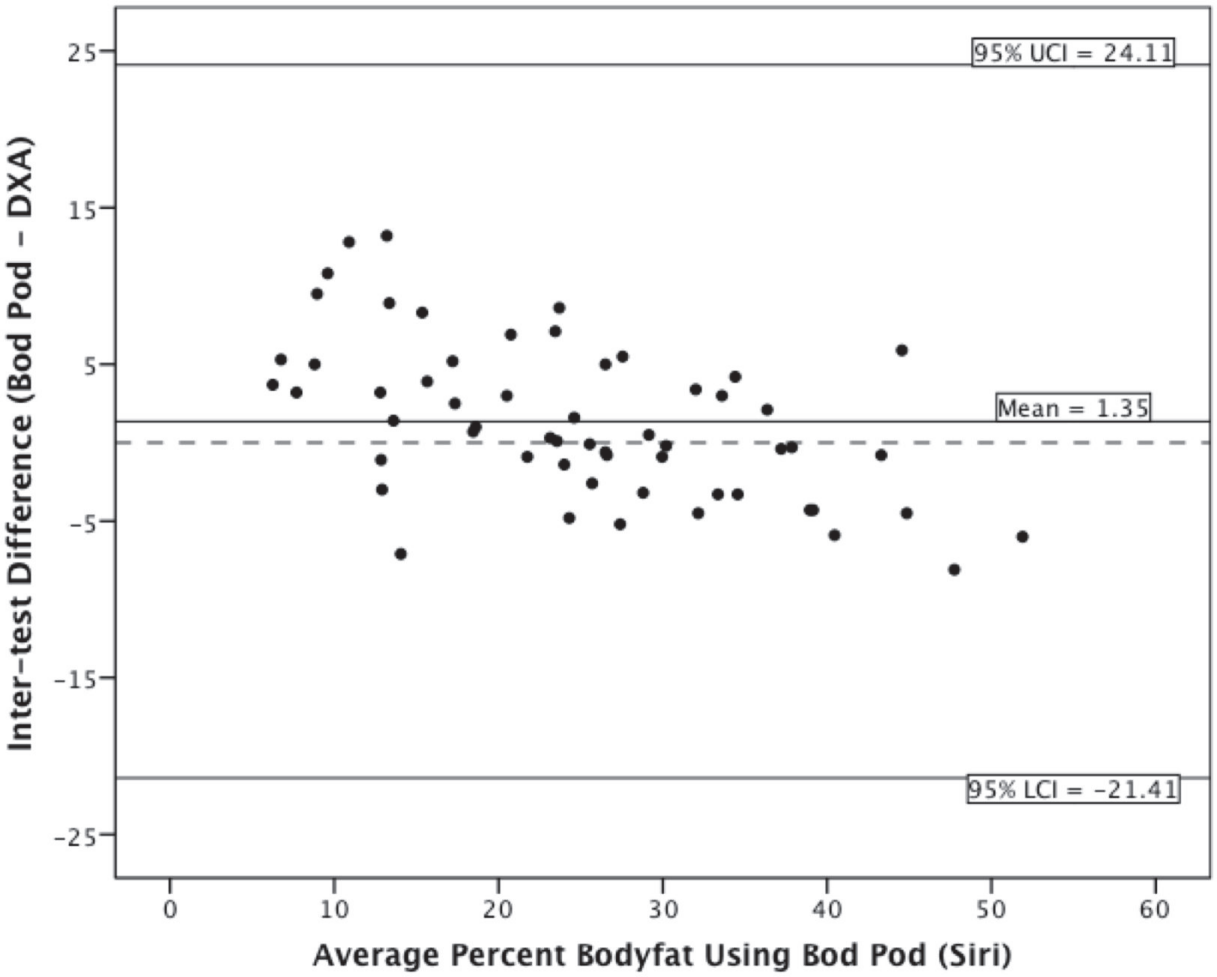
Bland-Altman plot displaying Siri estimates.

**Figure 2:**
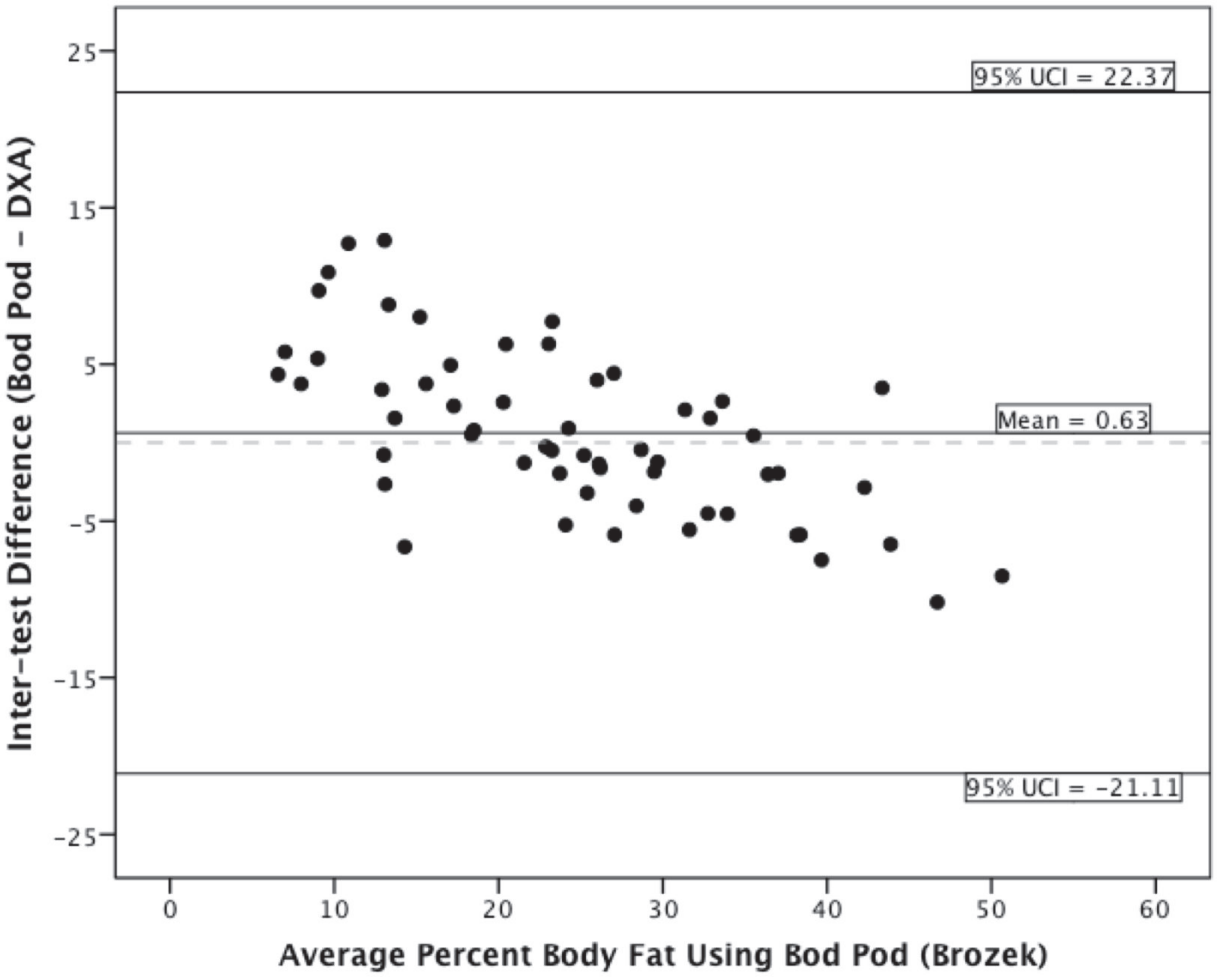
Bland-Altman plot displaying Brozek estimates.

The Bland-Altman plots for the entire sample appear in Fig. 1 (Siri estimates) and Fig. 2 (Brozek estimates). As the plots indicate, the Bod Pod appears to overestimate body fat percentage in thinner adults and underestimate body fat percentage in heavier adults, regardless of which equation we used. For the underweight group, the magnitude of the inter-test difference was larger than in the overweight/obese group. The Bod Pod and DXA had good agreement in the normal-weight individuals.

## Discussion

In this study, we compared DXA-derived estimates of body fat percentage to Bod Pod estimates in three BMI groups, and found that Bod Pod estimates tended to diverge from DXA estimates at the extremes of the BMI spectrum. The Bod Pod appeared to overestimate body fat percentage in thinner adults (up to 13.2%) and underestimate body fat percentage in heavier adults (up to −8.51%). The divergence appeared to be greater at lower percentages of body fat. Although some studies have examined ADP in obese subjects [9,10,14,15] and also find a relatively small divergence between ADP and DXA, this study is novel in that it contributes data regarding underweight subjects and finds the discrepancies to be larger in magnitude.

The difference in measurement between ADP and DXA may partially be explained by the assumptions that are inherent in the calculation of body fat percentage from direct measurements. The ADP body fat percentage calculations used a 2-compartment method. This assumes that the body is composed two tissue types: Fat and lean mass. The actual composition of fat-free mass includes bone, water, muscle, vasculature, connective tissue, and more. The degree of variance of fat-free mass is not well accounted for in this method. DXA can additionally determine bone density, thus eliminating one degree of variability and potentially making the measurement more accurate.

Additionally, Siri and Brozek are densiometry formulas designed to estimate body fat percentage from body density. Body fat percentages for extremes in body composition might not best be calculated by this general formula. There may be a need for specific formulas that can better assess body fat percentages in underweight.

A final possibility that might explain the pattern of findings is that the underlying assumption of constant hydration of lean body mass in DXA may have been violated. During the early phases of voluntary weight loss, constant hydration may not be assumed [16], an issue that may have differentially affected the calorie restrictors in the underweight group. However, only calorie restrictors with at least a 2-year history of calorie restriction were included in this study, suggesting that they were not in the early phase of voluntary weight loss. Whenever possible, we obtained third-party confirmation of this 2-year inclusion criterion from the leaders of the two organizations from which we recruited this group (Calorie Restriction Society: Vice President and Board member; CR Way: President and Executive Vice President). We therefore suspect that the differences observed between DXA and Bod Pod in the underweight group were not due to differences in hydration.

The Bod Pod is appealing because of its low subject burden, easy operation, and minimal costs for testing runs. The results from this study suggest, however, that in patient populations that are very thin, more precise methods may be warranted. If only the Bod Pod is available, then at minimum, techniques [17] to control measurement error in the Bod Pod (such as maintaining room temperature, voiding bladders, using appropriate clothing, etc.) should be followed carefully.

This study was limited by its small sample size and limited racial/ethnic diversity. As such, generalization should be made with caution, and the mean difference estimates we observed should not be used as correction factors. However, a notable strength of this study was its unique population of healthy but very thin participants. A second limitation is that we were not able to compare Bod Pod estimates to hydrostatic weighing. Here we relied on DXA, which is not without its own drawbacks and limits on accuracy [18], but which takes into account individual differences in bone density, making it more precise than the Bod Pod [19]. For this reason, we believe the differences observed are due to limitations of the Bod Pod technology rather than to anomalies in our sample selection. However, we acknowledge that given the unique nature of the participants, subject selection may have also influenced these results. Finally, we did not have repeated measures of each method, so we cannot speak to the magnitude of changes that can be detected by the Bod Pod in, for example, weight loss intervention studies (although others have found good test-retest reliability in the Bod Pod, e.g., *r* = .99 and no significant difference between repeated measures; [11]). This would be an interesting and fruitful future direction.

This study additionally contributes findings indicating the direction of bias in Bod Pod estimates. In other words, clinicians using the Bod Pod with lower BMI values might assume that these are on average likely upper limits of body fat percentage estimates, and vice versa, which may aid in treatment decision-making.
